# Emerging complexities of APOBEC3G action on immunity and viral fitness during HIV infection and treatment

**DOI:** 10.1186/1742-4690-9-35

**Published:** 2012-04-30

**Authors:** Mahdis Monajemi, Claire F Woodworth, Jessica Benkaroun, Michael Grant, Mani Larijani

**Affiliations:** 1 Immunology and Infectious Diseases Program, Division of Biomedical Sciences, Faculty of Medicine, Memorial University of Newfoundland, Newfoundland, Canada; 2 Mani Larijani, Division of Biomedical Sciences, Faculty of Medicine, Health Sciences Center, MUN, 300 Prince Phillip Dr., St. John’s, NL, A1B 3V6, Canada; 3 Division of Biomedical Sciences, Faculty of Medicine, Health Sciences Center, MUN, 300 Prince Phillip Dr., St. John’s, NL, A1B 3V6, Canada

## Abstract

The enzyme APOBEC3G (A3G) mutates the human immunodeficiency virus (HIV) genome by converting deoxycytidine (dC) to deoxyuridine (dU) on minus strand viral DNA during reverse transcription. A3G restricts viral propagation by degrading or incapacitating the coding ability of the HIV genome. Thus, this enzyme has been perceived as an innate immune barrier to viral replication whilst adaptive immunity responses escalate to effective levels. The discovery of A3G less than a decade ago led to the promise of new anti-viral therapies based on manipulation of its cellular expression and/or activity. The rationale for therapeutic approaches has been solidified by demonstration of the effectiveness of A3G in diminishing viral replication in cell culture systems of HIV infection, reports of its mutational footprint in virions from patients, and recognition of its unusually robust enzymatic potential in biochemical studies in vitro. Despite its effectiveness in various experimental systems, numerous recent studies have shown that the ability of A3G to combat HIV in the physiological setting is severely limited. In fact, it has become apparent that its mutational activity may actually enhance viral fitness by accelerating HIV evolution towards the evasion of both anti-viral drugs and the immune system. This body of work suggests that the role of A3G in HIV infection is more complex than heretofore appreciated and supports the hypothesis that HIV has evolved to exploit the action of this host factor. Here we present an overview of recent data that bring to light historical overestimation of A3G’s standing as a strictly anti-viral agent. We discuss the limitations of experimental systems used to assess its activities as well as caveats in data interpretation.

## The role of APOBEC3G in HIV restriction

APOBEC3G (A3G) is a recently discovered primate-specific member of the apolipoprotein B mRNA-editing enzyme, catalytic polypeptide-like editing complex family of cytidine deaminase enzymes with potential to inhibit propagation of the human immunodeficiency virus (HIV) [1,2]. The APOBEC family includes eleven members in humans: activation-induced cytidine deaminase (AID), APOBEC1, APOBEC2, APOBEC3A-H, and APOBEC4 [3,4]. These enzymes convert deoxycytidine (dC) to deoxyuridine (dU) in single stranded DNA (ssDNA) or RNA of human and viral genomes, thereby affecting a variety of physiological functions [5-7]. A3G was discovered through the study of heterokaryons generated between cells permissive and non-permissive to infection by virion infectivity factor (Vif)-deficient HIV that were used to determine the action of the HIV protein Vif [1,8,9]. A3G is primarily expressed in CD4^+^ T lymphocytes, macrophages, and dendritic cells, which are all the natural targets of HIV infection [2,10-14]; although expression in other tissues may be induced by interferon(s) [15-18]. A3G mutates dC in nascent viral minus strand DNA generated by reverse transcription [17-24] and preferentially deaminates dC in signature trinucleotides (CCC, TCC) often referred to as hotspots [6,19-21]. The resulting dUs can trigger DNA degradation through the action of DNA repair pathways, such as those involving uracil DNA glycosylase and apurinic-apyrimidinic endonuclease [25,26]. For viral genomes that evade destruction, the consequent deoxyguanosine (dG) to deoxyadenosine (dA) substitutions in plus strand DNA can alter reading frames, introduce premature translation termination codons, and/or produce mutated viral proteins [7,20-25]. In addition, A3G can disrupt propagation of HIV by binding viral RNA, interfering with the DNA strand transfer acrobatics of reverse transcription, physically blocking reverse transcriptase (RT), and obstructing integration into the host cell genome [24,26-31]. A3G has been shown to block RT activity by decreasing tRNA priming, competing for binding to templates, restricting strand transfer during reverse transcription, and direct binding [28,32,33]. Beyond the reverse transcription stage, incorporation of dU into minus strand DNA of the HIV genome has been shown to interfere with synthesis of the complementary plus strand [23]. These findings initially led to the notion that A3G can inhibit viral propagation through pathways dependent or independent of its deamination activity; however, many studies supporting deaminase-independent activities utilized A3G overexpression. It has recently been appreciated that with low level A3G expression, which may be a more accurate representation of the physiological case, deaminase activity is required for viral restriction [34-39]. While the relative contribution of deamination independent activities to viral restriction remains contentious, these may prove more relevant to the action of A3G in restricting endogenous non-long terminal repeat retrotransposons, such as long and short interspersed nuclear elements [40-45]. The anti-retroelement activity of A3G may represent a host strategy to protect its genome from the deleterious effects of transposable elements. A possible mechanism could involve the binding of A3G to retroelements resulting in blockage of their mobility [46].

The recent expansion of a single APOBEC3 gene in mice to seven (APOBEC3A-H) in primates and the relatively high divergence within APOBEC3 enzymes in primates are evidence for immense evolutionary pressure on the locus suggested to possibly be concomitant with the emergence of modern lentiviruses [3,4,47,48]. Conversely, the finding that the accelerated rate of A3G divergence predates modern lentiviruses, together with the lack of a clear correlation between human A3G polymorphisms and the progression of acquired immunodeficiency syndrome (AIDS), suggest that lentiviral pressure may be, at best, only partially responsible for expansion of the APOBEC3 locus [49-52]. This manner of growth in host defence capacity can reciprocally drive co-evolution of highly adaptable viruses. In this regard, we highlight an emerging body of evidence suggesting that the activity of A3G may be partially subverted by HIV for its survival benefit. These data support a more complex scenario in which the initial perception of A3G as a strictly anti-viral agent may have been naïve.

### Viral and cellular factors limiting APOBEC3G effectiveness

The view of A3G as a potent intrinsic anti-viral factor was largely borne out of findings of high levels of dG to dA hypermutated virus sequences in di- and tri-nucleotide motifs targeted by A3G [53-57]. In stark contrast, the previously recognized mutational machinery of HIV, RT, only introduces approximately one mutation per viral genome during a replication cycle [58]. Supporting the potency of A3G as a mutagenic agent is a wealth of biochemical data showing that it is a highly processive enzyme able to mediate multiple mutations on a given stretch of ssDNA. Accordingly, A3G significantly diminishes viral propagation in several cell culture experimental systems of HIV infection [7,20,22,42,47].

To counteract these activities, lentiviruses have evolved several strategies, primarily in the form of auxiliary proteins such as Vif, which binds and targets newly synthesized A3G for degradation via a ubiquitin-dependent proteosomal pathway [59-69]. A3G is packaged into virions in infected virus-producing cells and it has been shown that it is largely this virion-packaged fraction of A3G rather than the pool of cytoplasmic A3G that is most active on the viral genome in newly infected cells [70-74]. The number of A3G molecules incorporated into each virion is dependent on the level of A3G expression in the producer cell [75]. On average, 3 to 11 molecules of A3G are sufficient for effective viral restriction in the target cell [76]. Besides lowering A3G levels through degradation, Vif has also been suggested to directly interfere with A3G encapsidation and may impair its translation [66,74,77-80]. Vif utilizes other co-factors present in the target cell to ubiquitinate A3G and it was recently shown that Core binding factor (CBF)-β, a cellular transcription factor, is required for Vif-mediated degradation of A3G [81,82]. As a result, when Vif is present, the mutation levels induced by A3G and its effectiveness in viral restriction are diminished. That Vif is essential for HIV replication in A3G-expressing cells, and that the sole function of Vif was thought to be A3G inactivation, lent credence to the notion that A3G is a potent restrictor of HIV propagation [83]. On the other hand, it is now appreciated that even in the presence of Vif, A3G can still cause sub-lethal levels of dG to dA mutations [19,84]. It is possible that the preferential targeting of newly synthesized A3G by Vif leaves a fraction of previously synthesized A3G intact [85]. In addition, it appears that Vif expression does not completely abolish A3G activity and the correlation between the levels of viral infectivity and A3G inhibition by Vif is not absolute [62,78]. Other functions for Vif and Vif-mediated ubiquitination, besides A3G degradation, are also coming to light. For instance, along with the auxiliary protein Vpr, Vif can induce G2 cell-cycle arrest, which may contribute to CD4^+^ T lymphocyte depletion [86-89]. Vif thus mediates several functions that are independent of its interaction with A3G and is a variable negative regulator of A3G activity rather than a complete inhibitor.

A3G action is further limited by its entrapment in high-molecular-mass ribonuclear complexes (HMM) that may reach megadaltons in size, mediated by non-specific binding of cellular and/or viral RNA and proteins [12,71,90-97]. The shuttling of A3G into newly synthesized virions depends on binding viral RNA and/or proteins [98-101]. The requirement for high affinity interactions with RNA/DNA substrates may explain the evolution of A3G (and other APOBEC enzymes, e.g. AID) to contain an unusually high number of charged residues on its surface [102-104]. Ironically, this same attribute necessary to enact the anti-viral function of A3G may also be a key contributor to limiting its anti-viral function through HMM formation. Reversion of HMM to low-molecular-mass (LMM) A3G can be experimentally mediated by treatment with RNase A/H [70,71,105]. The RNase H activity of RT is thought to release viral RNA-bound A3G, allowing it to act on the proximal minus strand DNA during its synthesis [2,19,75]. Enzymatically active A3G able to be incorporate into newly synthesized virions is strictly found outside of the HMM complexes in the LMM fraction [73,106]. The LMM form primarily resides in peripheral blood-derived resting CD4^+^ T cells and monocytes [12]; however, upon activation of CD4^+^ T cells or differentiation of monocytes into macrophages, a higher proportion of A3G is shuttled to HMM complexes [2,91]. Although this was suggested to be a mechanism that restricted the infection of resting T cells by HIV, subsequent knockout studies of LMM A3G in resting CD4^+^ T cells did not render these cells permissive to HIV infection, thus indicating that the difference in the LMM- versus HMM-bound proportion of A3G is not the sole mechanism for resistance of resting CD4^+^ T cells to HIV infection [107,108]. Beyond the induction of HMM formation by HIV through cellular activation processes, Vif has been shown to directly promote HMM production [109]. Remarkably complex co-evolution is evident considering the intimate linkage between HIV infection and HMM formation and the notable level of mechanistic integration between A3G function and the viral replication machinery. The RNase H activity of RT is at once both necessary and detrimental to viral propagation due to its role in the release of active A3G.

The complexities surrounding regulation of Vif activity and HMM formation notwithstanding, it is clear that both result in diminished A3G efficacy. It is possible that mutations introduced by A3G only succeed in restricting viral replication at a sub-optimal level and conversely may assist the virus by generating sequence variation [35,39,84]. Consequently, an alternative view that A3G activity can contribute to viral fitness has recently gained strong support. In the following sections, we highlight evidence for the pro-viral activities of A3G. At the same time, we discuss caveats of experimental systems and data interpretation that must henceforth be considered in development of a revised and better-informed picture of A3G function.

### The role of APOBEC3G in generation of anti-viral drug resistant HIV

Gain of resistance to drug(s) used in the treatment of HIV is a major determinant of viral evolution during the course of disease. To date, almost a hundred drug resistance mutation sites have been identified in the HIV genome [110]. These induce resistance to common anti-HIV drugs acting as nucleoside/nucleotide analogue RT inhibitors, such as 2',3'-dideoxy-3'-thiacytidine (3TC), abacavir (ABC), and 2',3'-dideoxyinosine (DDI), as well as non-nucleoside/nucleotide analogue RT inhibitors, including Nevirapine (NVP), Delavirdine (DLV), and Efavirenz (EFV) [110]. Drug resistance mutations function directly by altering drug targets or indirectly by modifying pathways that contribute to drug escape. Many drug resistance mutations have been shown to reside in A3G hotspots [111].

A bioinformatics study assessed the probability of A3G mutations in known drug resistance sites taking into consideration the double-crested gradient of A3G-induced mutational levels throughout the HIV genome. Out of 52,000 G to A mutations, only 695 (1.3%) were located in drug resistance sites [112]. In this context, the investigators reported a modest correlation between A3G activity and the generation of drug resistance mutations relative to the overall footprint of A3G on the HIV genome [19,112,113]; however, recent experimental evidence more strongly implicates A3G in the generation of drug resistance mutations. For example, the very common M184I(V) mutation of RT that causes resistance to 3TC and, to a lesser extent, ABC and DDI, is located in an A3G hotspot (TCCAT to TCUAT) and is produced by A3G in vitro during HIV replication in cell culture systems [114]. Intriguingly, this was observed in the absence of 3TC in as many as 40% of sequenced proviruses, reflecting a pre-treatment pool of resistant viruses poised for propagation after drug exposure [29,115-118]. Because this mutation may in fact reduce viral replication fitness in the absence of 3TC [119-122], it is likely that this measurement actually underestimates the role of A3G in the generation of this mutation. In support of this notion, the M184I mutation emerges at significantly higher rates when the virus is grown in A3G-expressing as compared to A3G non-expressing host cells, indicating that A3G activity is the major source of this mutation [123]. This is a striking example of the parallel role of A3G in simultaneously aiding host and virus: in the same manner that it acts as a pre-existing innate immune factor that fortifies host defenses prior to viral exposure, A3G boosts the inherent ability of HIV to gain resistance even before drug treatment. That this mutation is associated with a decline in viral fitness may indicate that drug resistance presents a significant source of pressure in viral evolution resulting in the gain or maintenance of A3G hotspots in key positions in the viral genome.

If the contribution of A3G action to drug resistance and survival of HIV is a biologically considerable one, the evolution of HIV during disease could involve active relaxation of A3G inhibition. Indeed, direct evidence for this phenomenon was provided by the prevalence of the Vif K22H mutation in patients failing drug treatment, as compared to treatment-naïve patients [124,125]. Vif K22 is a key residue for interaction with A3G, and Vif K22H exhibits reduced effectiveness in neutralizing A3G [115]. Ex vivo infection of peripheral blood mononuclear cells (PBMCs) with viral stocks harboring various other Vif mutations that are unable to deactivate A3G (e.g. Vif K22E) yielded a significant increase in the generation of M184I mutants [114]. In addition, several drug resistance mutations, including M184I in RT and G16E/M36I in the protease, are significantly more common in patients harboring elevated relative levels of K22H-mutated viruses [125]. Like the M184I mutation, both G16E and M36I mutation sites are located in A3G hotspots. Thus, not only does HIV benefit from spontaneous pre-drug treatment A3G-induced mutations in a passive, somewhat random manner, it appears that resistance sites for some of the most commonly used drugs arose in A3G hotspots. This in no way implies viral sentience, but merely indicates a selective advantage derived from the overlap of sites more susceptible to mutation (A3G hotspots) being able to confer drug resistance.

### The contribution of APOBEC3G to the evasion of adaptive immunity by HIV

Restrictions imposed on the activity of A3G by Vif and HMM limit its effectiveness as an innate immune agent; however, following the first weeks of HIV infection, development of B and T cell mediated adaptive immunity partially controls viremia [116-118,126]. A central facet of the adaptive immune response is elimination of infected target cells by cytotoxic T cells (CTL), as highlighted by the close inverse association between robustness of the CTL response with viremia levels and disease progression [127-129]. Thus, evasion of the CTL response is thought to be a powerful driving force for the evolution of HIV during disease, as confirmed by several studies showing the prevalence of CTL escape in HIV infection [130,131]. CTL evasion may result from alterations in CTL access to infected cells. For instance, the auxiliary HIV protein Nef modulates class I MHC expression to decrease the recognition and killing of infected cells [132]. Alternatively, CTL evasion may result from alterations in the interactions between the CTL and infected target cell. Mutations in CTL recognition epitopes have been shown to mediate CTL evasion through modulating the efficacy of CTL activation [133-135].

As in the case of drug resistance, it is possible that HIV can exploit the limited non-lethal action of A3G to generate CTL escape mutants. In support of this model, a study examining CTL escape during early infection found that approximately a third of the rapidly mutating sites mediating CTL escape were embedded in A3G hotspots, with more highly mutating sites being relatively enriched in A3G hotspots [136]. Twenty-four rapidly diversifying sites were identified at which G to A mutations were 2–3 fold more frequent than the overall G to A mutation rate across the entire HIV genome (29 versus 12%). Fourteen of these sites located in or near CTL epitopes. These data suggest that it may be advantageous towards immune escape for HIV to maintain A3G hotspots in areas where mutations can affect processing, presentation or recognition of T cell epitopes, or conversely to establish T cell epitopes near A3G hotspots.

In contrast, another study reported that A3G mutations enhance the virus-specific CTL response through the introduction of premature stop codons into the HIV genome that cause the generation of truncated or misfolded proteins [137]. In this study, Vif+ or Vif- HIV was produced in the presence or absence of A3G in a cell line and subsequently used to infect PBMCs followed by assessing their susceptibility to MHC-matched peptide-specific CTL clones. It is possible that the finding of enhanced target cell killing as a result of A3G activity reflects an inherent bias of the specificity of the CTL clones examined. In addition, given the numbers, diversity, and relative scarcity of CTL specific for each particular peptide in vivo, the general biological relevance of this work remains to be determined. Therefore, although A3G appears to play a role beyond innate immunity and modulate adaptive immunity, further work is required to elucidate the nature and extent of this activity.

### Manipulation of APOBEC3G effectiveness: implications and challenges for the design of therapeutic approaches

To date, multiple avenues have been suggested and/or pursued towards exploitation of A3G as an antiviral therapy. These approaches include the development of small molecules that inhibit the interaction between Vif and A3G and/or inhibit interactions with cellular factors that act downstream of Vif, enhancement of LMM formation over HMM formation, and increasing A3G levels by treatment with interferons or gene-therapy delivery of A3G along with the restriction factor Trim 5α [70,138-140].

Strategies to down-regulate the action of Vif and HMM that were initially suggested as therapeutic approaches have recently been questioned in light of the increasingly apparent pro-viral activities of A3G [35,141]. Disturbing the Vif-APOBEC interaction presents a delicate challenge because subtle adjustments to Vif activity have been shown to modulate levels of A3G activity. For instance, naturally occurring patient-derived virions harboring Vif mutations selectively exhibit viral genome sequence variations consistent with survival advantage under their environmental pressures [25,54,125]. Incomplete Vif inhibition might increase effective A3G concentrations and in so doing actually accelerate viral evolution by only modestly increasing non-lethal mutation rates [22,111]. On the other hand, complete Vif inhibition may result in A3G activity levels high enough to tip the balance towards immunity through mutation loads capable of disabling viral replication. The isolation of viral sequences harboring Vif mutants significantly diminished in their ability to neutralize A3G challenges this scenario as it brings into question the ability of A3G to fully abrogate the propagation of Vif-deficient viruses [124,125]. Furthermore, it may also be important to consider the involvement of Vif inactivation in the generation of drug resistance as a cautionary note against therapies designed for complete elimination of Vif activity.

Studies of the effect of A3G expression levels on HIV disease progression rates in both humans and other primates have yielded conflicting results. One investigation reported an inverse correlation between A3G expression levels and disease progression [142,143], while another noted no such association [144]. A third study conducted on SIV-infected rhesus macaques reported an inverse correlation between A3G expression levels and disease progression [145]. Further work will be required to conclusively define any association between A3G expression patterns or levels and HIV disease progression. In addition, it is not clear whether A3G expression levels can influence the relative extent of its pro- versus anti-viral activities. If indeed there is any correlation, it remains to be determined where the threshold level of A3G activity lies and whether it varies during the course of infection. Whatever the pivotal point, the underlying premise that regulating A3G activity by modulating Vif/HMMs can alter viral mutation levels to the detriment of HIV may be flawed in viewing HIV as an acquiescent canvas for mutational activity. Examination of the spectrum of A3G-induced mutations during the viral life cycle paints a different picture in which there is a high level of mutation in viral DNA, an intermediate level in cellular RNA, and a low level in viral RNA. Non-advantageous or detrimental mutations are serially filtered out during the transcription, nuclear-cytoplasmic transport, translation and assembly phases of the viral life cycle, resulting in a pool of virions emerging from the host cell that bear a suppressed footprint of total A3G mutational activity – a process termed purifying selection [146]. Although at first glance it ought to decrease viral variation, purifying selection is balanced in favour of HIV by other diversification processes, such as recombination between mutated and wild-type viral genomes [147,148]. This is a sophisticated mechanism of protection for the virus as it enhances the potential for beneficial mutations to propagate quickly and represents a heretofore unappreciated layer of complexity when considering therapeutic strategies centered around modulating the activity and/or levels of A3G.

### The relative contribution of APOBEC3G in the context of other viral factors to HIV evolution

Formulating therapeutic strategies also requires a careful assessment of the relative contributions of non-A3G factors to the sequence variation of HIV. In general, retroviral genomes are prone to a high frequency of mutation [149-153]. The elevated error rate of the HIV RT, alterations in the nucleoside triphosphate (NTP) and deoxynucleotide triphosphate (dNTP) levels that affect polymerase accuracies, and the lack of proofreading machinery during viral genome replication all contribute to the highly mutagenic nature of viral genomes [58,151]. In addition to mutations, HIV exhibits a notably high rate of genomic recombination amongst retroviruses, possibly due to its cellular transmission properties resulting in frequent co-infection by genetic variants [154-157]. Unlike in humans, recombination in retroviruses does not result from breakage and rejoining of DNA, but is instead mediated by the ability of RT to switch templates between the two encapsidated proviral RNAs [158-160].

Distinguishing between the actions of RT versus A3G is essential in determining the relative contribution of each to HIV pathogenesis given that its genome is predominated by dA nucleotides, and dG to dA changes are key to the generation of many drug resistance variants [111,161,162]. Prior to the discovery of A3G, RT was viewed as the main generator of genetic diversification in the HIV genome throughout the course of infection; however, both RT and A3G most frequently induce dG to dA transition mutations on the plus viral DNA strand [163]. Although a degree of uncertainty arises in assigning the source of hypermutations in the HIV genome, the fact that A3G preferentially deaminates dC nucleotides in signature trinucleotide hotspots (CCC, TCC) can be used to assign mutations [6,19-21]. In contrast to RT, which is capable of introducing one to two mutations in each viral genome per replication cycle [58], A3G is a highly processive and robust deaminase enzyme [164]. The rate of dG to dA hypermutation found in HIV genomes is approximately 1000 fold higher than RT alone would be expected to introduce [71]. Furthermore, A3G-expressing cells support significantly more HIV hypermutation than their A3G-deficient counterparts. While the impact of A3G on mutational load is tempered during wild-type HIV infection by factors such as Vif and HMM, and potentially obscured from the circulating virus pool by purifying selection, these and other observations provide evidence that A3G can and does make substantial contributions to HIV sequence variation [123]. Somewhere between the unfettered A3G activity that causes a lethal mutational load and complete A3G inhibition by Vif and other cellular factors lies a level of activity with the potential to favor drug resistance, immune escape and viral fitness. Given the demonstrated ability of HIV to adapt to its host, it would be surprising if adaptations deriving benefit from some level of A3G activity have not occurred.

## Conclusions

**Figure 1:**
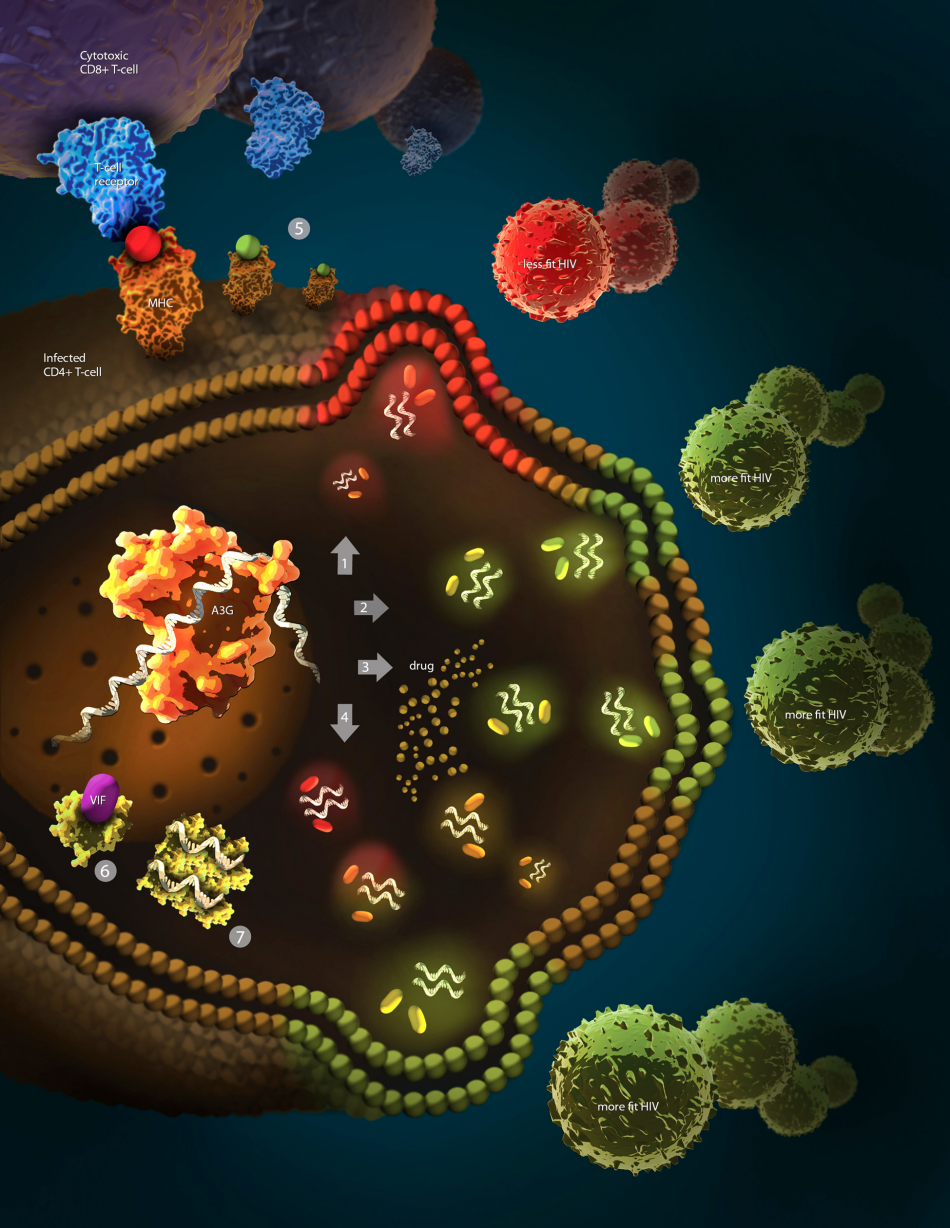
**The complexities of the pro- and anti-HIV actions of APOBEC3G.** Cross section into the cytoplasm of an infected CD4^+^ T cell is shown, with A3G (yellow) bound to the minus strand ssDNA of the viral genome (white). Virus is shown as green (fit virus) or red (unfit virus) circles forming within and budding out of the infected T cell. Each viral particle contains two copies of the RNA genome and multiple copies of A3G (rods). On the outside of the infected T cell, a cytotoxic CD8^+^ T cell (CTL) is shown recognizing a viral epitope in the context of MHC class I on the surface of the infected T cell. Arrows depict several possible outcomes of A3G action: (1) the classic mode of A3G action as an innate host defense agent whereby it generates mutations in the viral genome resulting in less fit or deactivated virions (red); (2) some low level mutations by A3G that may result in the production of more fit virions (green); (3) A3G may induce mutations in the viral genome that result in drug resistance, as shown by the emergence of more fit virions (green) through the pool of cytoplasmic drug (yellow dots); (4) the process of purifying selection wherein a heavy mutation load on the viral genome is filtered out throughout various stages in the viral life-cycle, resulting in selection for a final pool of viruses with low level mutations that may enhance viral fitness; (5) the mutations generated by A3G may result in the alteration of MHC class I-restricted viral peptide epiotpes such that recognition by CTL is abboragated (A3G-mutated CTL escape epitopes that result in the cloaking of the infected cell from the CTL response are shown in green while wild-type CTL epitopes that result in the recognition and killing of the infected cell are shown in red); (6) the virion infectivity factor (Vif) of HIV (purple) binds cytoplasmic A3G marking it for degredation; and (7) cytoplasmic A3G is trapped in high-molecular-mass (HMM) ribonuclear complexes and consequently rendered ineffective.

Figure 1 illustrates the various topics discussed herein with respect to the dual role of A3G in aiding the host or virus. As shown, there are clear instances when HIV can take advantage of A3G-induced mutation across a range of activity levels. Low mutation rates do not inactivate the viral genome and may in fact contribute to both drug resistance and immune escape. Conversely, HIV genomes suffering high mutation rates may be filtered out during viral replication to favor viral progeny with better fitness. Therefore, an ominous picture emerges wherein regardless of the action of A3G, HIV gradually gains the upper hand as a result of its fast replication rate and purifying selection processes that allow it to essentially optimize A3G mutation loads in progeny virions and better adapt to host defences and other selective pressures.

On the other hand, interpretation of studies examining the effect of A3G on the drug and CTL escape mutations in the HIV genome is subject to a major caveat. To date, studies identifying CTL escape or drug resistance mutations have been conducted using two general approaches: firstly, by searching for such mutations in clinical isolates; and secondly, by analyzing mutations in cell culture infection systems where A3G is expressed. We suggest that these types of studies are inherently biased towards generating the observed results and missing the bigger picture. In the first case, the virus pool obtained from infected individuals will inevitably be enriched for CTL and drug escape mutants as these have a replication advantage wherein virions harboring CTL or drug target motifs modified by A3G in a way that supports the opposite outcome (i.e. enhanced CTL recognition or increased drug susceptibility) would have been efficiently eliminated. Therefore, A3G could potentially create new or more immunogenic epitopes that have not yet been characterized. Furthermore, any suggestion that CTL escape is merely serendipitous neglects the point that a limited number of high quality escape epitopes are selected, as opposed to a large quantity of epitopes with low immune evading potential or substantial negative effects on viral fitness. HIV features a very economical propagation process in that the cost of having some genes manipulated is in-turn compensated for by a net effect favoring evasion of composite selective pressure. In the case of cell culture systems examining the role of A3G in generating drug resistance variants, the same caveat stands. That is, multiple drug resistance mutations have been identified and well characterized due to their prominence in patients. A3G-induced mutations that may conversely enhance drug susceptibility have not been identified because of their scarcity caused by more rapid elimination. Accordingly, we suggest that instances where A3G may in fact bestow the upper hand upon the host by generating mutations that enhance CTL recognition or drug susceptibility have likely been underestimated because of their inevitable transience. It is probable that the pro- and anti-viral activities of A3G are not mutually exclusive and that, at different points throughout HIV infection and in different patients, both scenarios unfold; however, the principles of purifying selection are active at the level of individual cells and that of the entire host organism, which buries the evidence of maladaptive A3G-imposed mutations beneath an avalanche of fast replicating adapted variants. While new experimental approaches are required to identify the relative proportion of both categories of A3G-induced mutations in an unbiased manner, the final outcome following multiple selection processes will determine the global impact of A3G mutations. Even if thousands of A3G-induced mutations favoring the host occur for a single mutation that favors HIV, the net advantage will be to HIV as long as one favorable mutation becomes incorporated into the circulating viral pool. Thus, the overall context within which A3G acts is probably just as relevant as the ratio of pro- versus anti-viral mutations. Resolution of this bigger picture will be critical in order to guide future therapeutic strategies centered on altering A3G activity.

## Competing interests

Authors do not have any competing interests.

## Authors’ contributions

MM and CW analyzed the literature and drafted the manuscript. JB contributed to the introduction. MG and ML prepared and edited the manuscript. All authors read and approved the final manuscript.
